# Em Busca de uma Previsão Pragmática de Risco na Insuficiência Cardíaca

**DOI:** 10.36660/abc.20250685

**Published:** 2025-12-11

**Authors:** Tiago A. Vaz, Nadine Clausell

**Affiliations:** 1 Instituto de Cardiologia Porto Alegre RS Brasil Instituto de Cardiologia, Porto Alegre, RS – Brasil; 2 Hospital de Clínicas de Porto Alegre Porto Alegre RS Brasil Hospital de Clínicas de Porto Alegre, Porto Alegre, RS – Brasil

**Keywords:** Insuficiência Cardíaca, Inteligência Artificial

A estratificação de risco para insuficiência cardíaca aguda (ICA) busca um equilíbrio delicado: os modelos devem ser suficientemente precisos para orientar as decisões, mas também simples e acessíveis para uso generalizado. Nesta edição dos Arquivos Brasileiros de Cardiologia, Ferreira et al. apresentam o ML-HF Score, uma ferramenta de aprendizado de máquina projetada para prever a mortalidade hospitalar em ICA, que aplica uma filosofia clássica da ciência de dados médicos, exemplificada pelo estudo seminal de Robert Detrano, de 1989, sobre doença arterial coronariana, utilizando algoritmos que superam os métodos existentes.^1,2^

Como prova da importância da validação em populações reais, o trabalho de Detrano demonstrou que características clínicas e não invasivas, cuidadosamente selecionadas e de baixo custo, podem alcançar precisão diagnóstica comparável a abordagens mais complexas. O conjunto de dados resultante, posteriormente arquivado no Repositório de Aprendizado de Máquina da UCI, se tornou fundamental para a avaliação comparativa de algoritmos, comprovando que modelos melhores podem ser construídos com menos recursos e mais acessíveis, desde que sejam validados em diversas populações.^2-4^

Ferreira et al. aplicam essa filosofia ao desafio contemporâneo da ICA.^1^ Utilizando dados do programa nacional Boas Práticas em Cardiologia (BPC), que inclui hospitais públicos terciários nas cinco macrorregiões brasileiras,^5^ os autores desenvolveram uma ferramenta preditiva utilizando um algoritmo Random Forest explicável. Após a aplicação do método Boruta para seleção de variáveis, 17 variáveis foram retidas, abrangendo desfechos clínicos, laboratoriais e relatados pelos pacientes.

Talvez a descoberta mais surpreendente tenha sido a proeminência do domínio de saúde física do questionário WHOQOL-BREF como o preditor mais forte de mortalidade, superando os parâmetros tradicionais. Essa inovação enfatiza os resultados relatados pelos pacientes (PROs) e está alinhada com as recomendações recentes das diretrizes para incorporar as perspectivas dos pacientes na avaliação de risco. Além dos PROs, medidas laboratoriais como sódio, creatinina e ureia, e características como pressão arterial sistólica, contribuíram substancialmente para o desempenho do modelo.^1^

Diversos pontos fortes merecem reconhecimento. A predominância do domínio de saúde física do WHOQOL-BREF destaca a importância de "escutar o paciente" sistematicamente. A metodologia seguiu as diretrizes TRIPOD e assegurou a validação interna e externa no Brasil.^1^ O escore ML-HF apresentou excelente discriminação no treinamento e manteve desempenho moderado na validação externa, superando os escores ADHERE e GWTG-HF.^1^

Entre as limitações, os autores reconhecem que a falta de dados pode ter introduzido viés de seleção e limitado a generalização. Subpopulações específicas podem ser afetadas na calibração, o que significa que o modelo pode precisar de ajustes locais em diferentes contextos. Alguns grupos de pacientes, como aqueles transferidos para outros hospitais, inscritos para transplante, com suporte de dispositivos de assistência ventricular ou que faleceram nas primeiras 24 horas, foram excluídos, o que é razoável do ponto de vista metodológico, mas significa que o modelo pode ser menos representativo dos casos mais graves ou complexos de insuficiência cardíaca. Por fim, a implementação prática também pode ser desafiadora, pois requer infraestrutura computacional, e o WHOQOL-BREF pode ser difícil de administrar em emergências.

Uma camada adicional envolve o custo. Nos laboratórios privados brasileiros, os custos dos testes de BNP ou NT-proBNP permanecem heterogêneos, com preços muito mais altos em laboratórios privados em comparação com os reembolsos. Essa discrepância destaca a tensão entre a utilidade clínica e as restrições financeiras, ajudando a explicar por que os testes de rotina permanecem indisponíveis, apesar das recomendações para ampliar sua adoção.^4^ Nesse sentido, a independência do escore ML-HF em relação a esses biomarcadores não é apenas uma limitação, mas uma adaptação necessária e estratégica às realidades econômicas, aumentando a viabilidade prática em diversas regiões do Brasil.

Ao lado do trabalho de Detrano, o ML-HF Score ilustra a continuidade e a inovação na análise de dados em saúde: ambas as iniciativas visam antecipar riscos utilizando os dados mais acessíveis e adequados ao contexto disponíveis. Detrano demonstrou que modelos diagnósticos robustos podem ser construídos a partir de características clínicas simples;^2^ Ferreira et al. demonstram que, utilizando Inteligência Artificial, é possível identificar novos preditores de risco de baixo custo, incluindo PROs. Ambos os esforços nos lembram que o melhor modelo não é apenas o mais preciso, mas também o mais utilizável em contextos clínicos reais.^1^

Em conclusão, o escore ML-HF representa uma importante contribuição para o cuidado da insuficiência cardíaca no Brasil. Ele demonstra como refinar a estratificação de risco, levando em consideração as limitações de recursos. Ao buscar algoritmos modernos com menos recursos, o estudo destaca que a inovação na medicina se baseia na praticidade, na equidade e na escuta do paciente. O próximo passo crucial será integrar esses modelos aos fluxos de trabalho clínicos e testar prospectivamente seu impacto na tomada de decisões e nos desfechos dos pacientes.^5^
