# Supplementary material for: Em Busca de uma Previsão Pragmática de Risco na Insuficiência Cardíaca
Source: Arq Bras Cardiol. 2025 Dec 11;122(11):e20250685. [Article in Portuguese] doi: 10.36660/abc.20250685 (PMC12978278; doi:10.36660/abc.20250685)
Supplement: Translation

# Translation: Pursuing Pragmatic Risk Prediction in Heart Failure

**DOI:** 10.36660/abc.20250685i

**Authors:** Tiago A. Vaz, Nadine Clausell

**Affiliations:** 1 Porto Alegre RS Brazil Instituto de Cardiologia, Porto Alegre, RS – Brazil; 2 Porto Alegre RS Brazil Hospital de Clínicas de Porto Alegre, Porto Alegre, RS – Brazil

**Keywords:** Heart Failure, Artificial Intelligence

Risk stratification for acute heart failure (AHF) pursues a delicate balance: models must be accurate enough to guide decisions, yet simple and affordable for widespread use. In this issue of *Arquivos Brasileiros de Cardiologia*, Ferreira et al. present the ML-HF Score, a machine-learning tool designed to predict in-hospital mortality in AHF that applies a classic philosophy in medical data science, exemplified by Robert Detrano's seminal 1989 study in coronary artery disease using algorithms that outperform existing methods.^1,2^

A testament to the importance of validation in real-world populations, Detrano's work demonstrated that carefully selected, inexpensive clinical and non-invasive features could achieve diagnostic accuracy comparable to more complex approaches. The resulting dataset, later archived in the UCI Machine Learning Repository, became a cornerstone for benchmarking algorithms, proving that better models can be built with fewer, more accessible resources, provided they are validated across diverse populations.^2-4^

Ferreira et al. bring this philosophy to the contemporary challenge of AHF.^1^ Utilizing data from the nationwide Best Practice in Cardiology (BPC) program, which includes tertiary public hospitals across all five Brazilian macro-regions,^5^ the authors developed a predictive tool using an explainable Random Forest algorithm. After applying the Boruta method for feature selection, 17 variables were retained, spanning clinical, laboratory, and patient-reported outcomes.

Perhaps the most striking finding was the prominence of the physical health domain of the WHOQOL-BREF questionnaire as the strongest predictor of mortality, surpassing traditional parameters. This innovation emphasizes patient-reported outcomes (PROs) and aligns with recent guideline recommendations to incorporate patient perspectives into risk assessment. Alongside PROs, laboratory measures such as sodium, creatinine, and urea, and features like systolic blood pressure, contributed substantially to model performance.^1^

Several strengths deserve recognition. The dominance of the WHOQOL-BREF physical health domain highlights the value of systematically "listening to the patient." The methodology followed TRIPOD guidelines and ensured both internal and external validation in Brazil.^1^ The ML-HF Score achieved excellent discrimination in the training and maintained attenuated performance in external validation, surpassing ADHERE and GWTG-HF scores.^1^

Among the limitations, the authors acknowledge that missing data may have introduced selection bias and limited generability. Specific sub-populations could be affected in calibration, meaning the model may need local tuning in different settings. Some patient groups, such as those transferred to other hospitals, listed for transplant, supported with ventricular assist devices, or who died within the first 24 hours, were excluded, which is reasonable from a methodological standpoint but means the model may be less representative of the sickest or most complex cases of heart failure. Finally, practical implementation may also be challenging, as it requires computational infrastructure, and the WHOQOL-BREF may be difficult to administer in emergency settings.

An additional layer involves cost. In Brazilian private laboratories, costs of BNP or NT-proBNP testing in Brazil remain heterogeneous, with much higher prices in private labs compared to reimbursements. This discrepancy highlights the tension between clinical utility and financial constraints, helping to explain why routine testing remains unavailable, despite recommendations to expand adoption.^4^ In this sense, the ML-HF Score's independence from such biomarkers is not merely a limitation, but a necessary and strategic adaptation to economic realities, increasing real-world feasibility in diverse regions in Brazil.

Placed alongside Detrano's work, the ML-HF Score illustrates continuity and innovation in healthcare analytics: both initiatives aim to deliver anticipation using the most accessible and context-appropriate data available. Detrano showed that robust diagnostic models could be built from simple clinical features;^2^ Ferreira et al. demonstrate that using Artificial Intelligence, it is possible to identify low-cost novel risk predictors, including PROs. Both efforts remind us that the best model is not only the most accurate but also the most usable in real clinical contexts.^1^

In conclusion, the ML-HF Score is an important contribution to heart failure care in Brazil. It demonstrates how to refine risk stratification while accommodating resource constraints. By seeking modern algorithms with fewer resources, the study highlights that innovation in medicine is about practicality, equity, and listening to the patient. The crucial next step will be to integrate such models into clinical workflows and prospectively test their impact on decision-making and patient outcomes.^5^
